# Low grade epithelial stromal tumour of the seminal vesicle

**DOI:** 10.1186/1477-7819-6-101

**Published:** 2008-09-23

**Authors:** Bruno Monica, Michelangelo Larosa, Francesco Facchini, Gianluigi Pozzoli, Ilaria Franceschetti, Irene Piscioli

**Affiliations:** 1 Division of Urology, Guastalla Hospital, Guastalla (RE), Italy; 2 Institute of Anatomic Pathology, S. Maria del Carmine Hospital, Rovereto (TN), Italy; 3 Department of Radiology, Budrio Hospital, Budrio (BO), Italy

## Abstract

**Background:**

The mixed epithelial stromal tumour is morphologically characterised by a mixture of solid and cystic areas consisting of a biphasic proliferation of glands admixed with solid areas of spindle cells with variable cellularity and growth patterns. In previous reports the seminal vesicle cystoadenoma was either considered a synonym of or misdiagnosed as mixed epithelial stromal tumour. The recent World Health Organisation Classification of Tumours considered the two lesions as two distinct neoplasms. This work is aimed to present the low-grade epithelial stromal tumour case and the review of the literature to the extent of establishing the true frequency of the neoplasm.

**Case presentation:**

We describe a low-grade epithelial stromal tumour of the seminal vesicle in a 50-year-old man. Computed tomography showed a 9 × 4.5 cm pelvic mass in the side of the seminal vesicle displacing the prostate and the urinary bladder. Magnetic resonance was able to define tissue planes between the lesion and the adjacent structures and provided useful information for an accurate conservative laparotomic surgical approach. The histology revealed biphasic proliferation of benign glands admixed with stromal cellularity, with focal atypia. After 26 months after the excision the patient is still alive with no evidence of disease.

**Conclusion:**

Cystoadenoma and mixed epithelial stromal tumour of seminal vesicle are two distinct pathological entities with different histological features and clinical outcome. Due to the unavailability of accurate prognostic parameters, the prediction of the potential biological evolution of mixed epithelial stromal tumour is still difficult. In our case magnetic resonance imaging was able to avoid an exploratory laparotomy and to establish an accurate conservative surgical treatment of the tumour.

## Background

The mixed epithelial and stromal tumour (MEST) is morphologically characterised by a mixture of solid and cystic areas with a biphasic proliferation of glands admixed with solid areas of spindle cells, with variable cellularity and growth patterns. We report a low grade MEST of the seminal vesicle and we emphasize the diagnostic criteria and the different diagnosis as cystoadenoma.

## Case presentation

**Figure 1:**
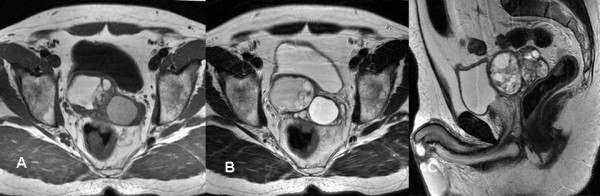
**Axial spin-echo T1-weighted (a) and axial (b) and sagittal T2-weighted (c) MRI showed a large, well-defined, multilocular pelvic mass in the side of the seminal vesicles, contiguous to the posterior wall of the urinary bladder and the prostate.** On T1 and T2-weighted images the mass showed internal septations that delimited heterogeneous iso- hyperintense areas with proteinaceus content.

**Figure 2:**
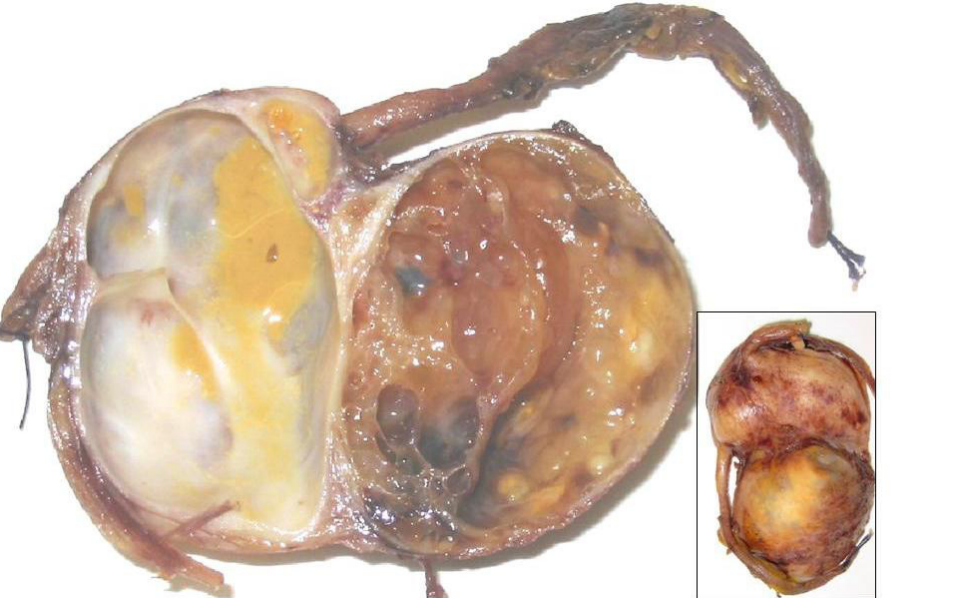
**The cut surface showed multilocular cists of varying size and shapes. **The segments of both the right and the left vas deferens were evident. Inset: the external surface was yellow, smooth and glistening.

**Figure 3:**
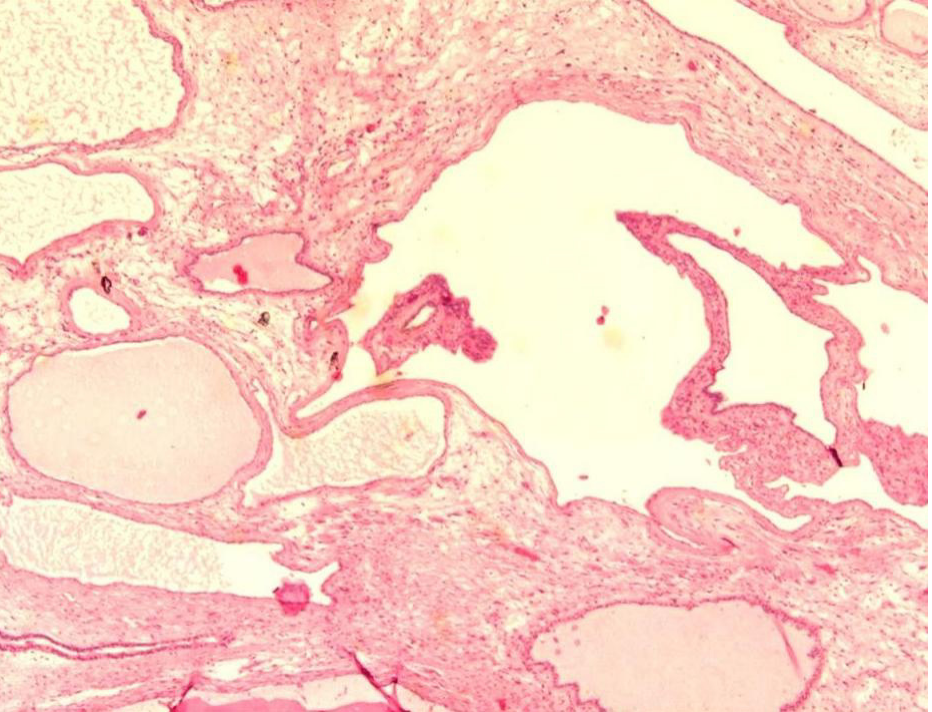
Microscopically the tumour showed cistically dilatated glands containing pale eosinophilic intraluminal secretions and lined by one to two layers of cuboidal or low columnar cells (H&E ×40).

**Figure 4:**
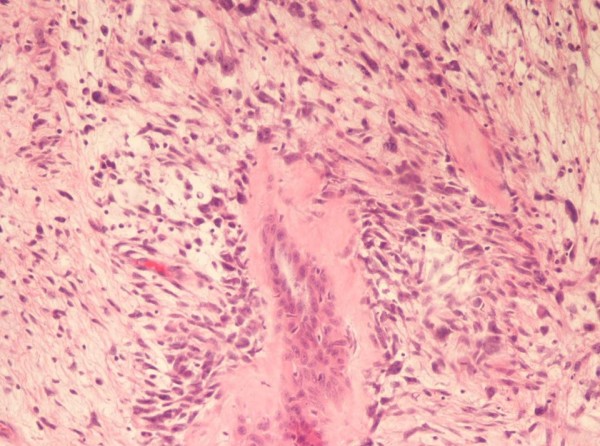
**The stromal cells were spindle-shaped and showed pleomorphism.** The stroma was at least focally densely cellular and tended to condense around distorted glands (H&E ×200).

**Figure 5:**
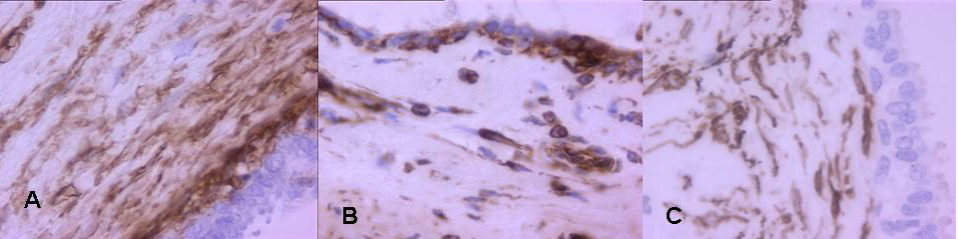
The stromal cells show positivity for AML (a)(×400), Vimentin (b)(×600) and CD 34 (c)(×600).

A 50-year-old man was admitted in November 2005 to our hospital with disuria, fever and gradual decrease in urinary stream for several months. On ultrasound (US) a hynomogeneous, hypoechoic pelvic mass of the posterior side of the bladder was demonstrated. Abdominal computed tomography (CT) revealed a 9 × 4.5 cm pelvic mass in the side of the seminal vesicles displacing the prostate and urinary bladder. On precontrast CT, the mass showed fluid heterogeneous content, well-defined margins, and irregular thin enhanced internal septa after intravenous contrast material administration. On axial spin-echo T1-weighted and axial and sagittal T2-weighted (Fig. 1a–c) magnetic resonance imaging (MRI), a large well defined multilocular pelvic mass was revealed in the side of the seminal vesicles, contiguous to the posterior wall of the urinary bladder, above the prostate and anterior to the rectum. On T1 and T2-weighted images, the mass showed internal septa that delimited heterogeneous iso- hyperintense areas with proteinaceus content. On axial T1-weighted fat-suppression gradient-echo images, before and after intravenous administration of gadopentetate-dimeglumine (gadolinium-DTPA), no enhancement of the mass occurred. The exploratory laparotomy revealed a retrovesical mass, which was well defined and not firmly adherent anteriorly to the bladder. It was supposed that the origin of the mass might be the right seminal vesicle. The tumour was totally dissected from the attachments to the bladder anteriorly ad rectum posteriorly, which required the removal of the left seminal vesicle and a portion of both vasa deferens. 14 months after surgery, the patient is currently alive with no evidence of disease. The tumour measured 9 × 6 × 6 cm and consisted of an oval rubbery mass. The cut surfaces showed multilocular cysts of variable sizes and shapes filled with gelatinous material (Fig. 2). The histological examination revealed two distinctive but intermixed components, one glandular and the other stromal (Fig. 3). The glandular proliferation was characterized by cystically dilated glandular spaces, containing pale eosinophilic intraluminal secretions and lined by one to two layers of cuboidal or low columnar cells. There was either no significant cytologic atypia or appreciable mitotic activity. The epithelial cells were uniformly reactive against all keratin proteins (AE1/AE3, CAM5.2, and high-molecular-weight keratin). Monoclonal and polyclonal carcinoembryonic antigen (CEA), prostate acid phosphatase (PAP) and prostate – specific antigen (PSA) stains gave negative results. The stromal component was composed by extensive loosely cellular areas with mixoid content. The stromal cells were spindle-shaped and showed pleomorphism. The stroma was at least focally densely cellular and tended to condense around distorted glands. No mitoses were found (Fig. 4). The spindle-shaped cells showed strong reactivity for vimentin, CD34, and patchy weaker reactivity (30%) for α-smooth muscle actin (Fig. 5a–c) and desmin, but were negative for cytokeratin and PSA. We preferred a diagnosis of low grade MEST because of the presence of cellular pleomorphism of the stromal component. 26 months after surgery, the patient is still alive, with no evidence of disease.

## Discussion

In our case the low-grade MEST diagnosis was made in accordance with the World Health Organization (WHO) classification criteria [1]. The lesion arose from the seminal vesicle and did not: a) present normal tissue, b) invade the prostate, and it was not immunoreactive for monoclonal and polyclonal CEA, PAP and PSA. The glandular proliferation was benign. Mitosis and atypia of the epithelium were not found. The stromal cellularity was mostly pronounced in the tissue adjacent to intratumoural glands. Focal cellular pleomorphism was present, but mitoses were not found. The examined lesion was categorized into low-grade MEST because of the presence of atypia in the spindle-shaped cells. The former literature reviews reported MEST under different names: cystomyoma [2], cystadenoma [3-12], mesonephric hamartoma [13], papillary cystadenoma [14], cystic epithelial-stromal tumour [15], mullerian adenosarcoma-like tumour [16], cystosarcoma phyllodes [17,18], phyllodes tumour [19], epithelial stromal tumour with phyllodes tumour-like features [20], benign fibroepithelial tumour [21].

These tumours have been considered to represent morphological variants of the same neoplasm, which may reveal local recurrence but not malignant transformation [7,10]. In the WHO classification [1], the seminal vesicle cystoadenoma (SVC) was excluded from the category of MESTs and considered as a distinct neoplasm; it should be histologically distinguished from MEST by its non-neoplastic stroma. Consequently the previous reports of MESTs have been critically revised (table 1). In the reviews of 10 and 14 MESTs, Baschinsky *et al*., [9] and Son *et al*., [19] reported respectively 6 and 8 cases of SVC. According to WHO criteria, the cases of SVC reported by Lagalla *et al*., [8], Bullock et al [6], Damjanov and Apic [22], Lundhus *et al*., [23], should be excluded from MESTs. This is because the description of stromal component is absent [6] or because microscopically they showed a non-neoplastic stromal component, that is typical of benign glandular tumours. The SVC described by Soule and Dockerty [3], Baschinky *et al*., [9], Mazzucchelli *et al*., [7], Gil *et al*., [11], the mesonephric hamartoma of Kinas *et al*., [13], the benign fibroepithelial of Zanetti *et al*., [20], should be considered MEST, because all these tumours showed simultaneous ductal and stromal proliferations. In the histological grading of stromal component, the WHO classification categorised MESTs in low or high grade, depending on mitotic activity and necrosis [1]. But the number of mitosis is not specified. In the high-grade MEST the stroma should be at least focally densely cellular and condensed around distorted glands. In the differential diagnosis between low and high-grade MEST the histological features reported by WHO classification are not exhaustive to achieve a conclusive diagnosis. The WHO rejected the concept of phylloides tumour despite the evidence that it is a separate entity from cystadenoma. In the MEST review of Son *et al*., [19] and Hoshi *et al*., [20] the classification of Baschinsky *et al*., [9] in low, intermediate, high-grade MEST was reported. Surprisingly this distinction is not described in the original paper of Baschinsky *et al*., [11]. In the description of cystosarcoma phyllodes, Fain *et al*., [17] examined the tumours reported by Mazur *et al*., [15] and Soule and Dockerty [3]: obviously the spectrum of phyllodes tumour reported by Fain *et al*., may be not accepted as conclusive because only three cases were considered. In our case US was only useful to detect the lesion, but both its site and relations with adjacent organs were not established. CT confirmed the presence, size, location, internal consistency, extension of the primary tumour, and the absence of distant metastases. MRI was useful in establishing the tumour origin from the seminal vesicles and its relations with the adjacent organs (Fig. 1). MRI with fat-suppression and administration of paramagnetic contrast agent is recommended to demonstrate the absence of tumour vascularisation and to indicate the benign non-vascular nature of the mass.

**Table 1:** Literature review of mixed epithelial and stromal tumour of seminal vesicle

**Authors**	**Years Presentation symptoms**	**Radiological findings**	**Surgical specimen/Treatment (symbol)**	**Histology/Follow up**
Plaut et al (1944)^	66. Asymptomatic Palpable mass in left lower abdominal quadrant	Not performed	14 × 11 × 8 cm mass connected to another 8.5 × 6 × 6 cm mass by a pedicle-like structure/*	Cystomyoma/NED 5 months after surgery
Soule H et al (1951)	47. Fatigue, nocturia, rectal mass on physical examination	Not performed	14 × 6 × 6 cm cystic mass/*	Cystoadenoma/Not reported
Kinas et al (1987)	63. Pelvic mass on physical examination	Pelvic mass compressing extrinsically UB (IVP), displacing the rectosigmoid to the left and upwards (barium enema). CT: large, soft tissue density located on the posterolateral aspect of the UB. The UB and rectum were displaced toward the left side without signs of invasion	Not reported/*	Mesonephric hamartoma/Not reported
Mazur et al (1987)	49. Acute urinary retention	IVP: 7.5 × 5.0 cm mass indenting the posterior and interior aspect of the UB	7 × 5 × 2.5 cm cystic mass at the first operation and 8.5 × 7 × 7 cm cystic mass at the second exploration/*=	Cystic epithelial stromal tumor/Recurrence locally 2 years after the first excision. Ned.18 months after re-excision
Fain et al (1992)	61. Acute urinary retention	CT: solid mass of high density in the region of the left SV	8 × 5 × 6.5 cm tan polypoid mass obliterating the left SV/°	Cystosarcoma phyllodes/Lung metastases 4 years after resection
Laurila et al (1992)	49. Gradual decrease in urinary stream for several years	Large fluid filled mass in the lower abdomen (US) located directly superior to the prostate and dorsal to the UB replacing the right SV (CT)	6 ×5 × 5 cm cystic mass/°	Müllerian adenosarcomalike tumour/NED 4 years after surgery
Mazzuc-chelli et al (1992)	63. Intermittent increasing pain in the left inguinal area	IVP: left external compression of the UB	3 × 1.5 × 1 cm mass located within the left SV/*Ω	Cystoadenoma (benign fibroepithelial and cystic tumour)/NED 8 years after surgery
Baschinsky et al (1998)	37. Bladder outlet obstruction and hematospermia	CT: 6.2 × 6.2 cm mass of mixed attenuation located posterior to the UB and anterior to the rectum	6.5 ×5 × 3.5 cm tumour with a coarsely lobulated, almost cerebriform contour and a smooth, glistening, tan surface/°#	Cystoadenoma/NED 6 months after surgery
Santos et al (2001)	49. lower abdominal discomfort	CT: 15 × 9.5 × 7 cm heterogeneous soft tissue density mass within the pelvis near the midline, situated in close proximity to the right of SV and anterior wall of the rectum	16 × 11 × 7 cm, well-circumscribed, oval, firm to rubbery solid-cystic mass/*	Cystoadenoma/Not reported
Abe et al (2002)	65. urinary hesitancy, frequency, and constipation	CT: 5.5 × 6 cm solid mass involving nearly the entire right SV, compressing the prostate to the left anterior side, but distinct from the prostate. IVP: compression of the UB to the left anterior side	Not reported	Cystosarcoma phyllodes/lung metastasis seven months after surgery, death 11 months after surgery
Gil et al (2003)	49. Asymptomatic	CT-MRI: 9 cm well-defined expansive tumour, predominantly cystic, with septations, replacing the left SV	7 × 5 × 4.5 cm cystic mass/*	Cystoadenoma/NED 3 years after surgery
Zanetti et al (2003)	62. Soft mass in the site of the left SV on rectal examination	US-CT: on the left retrovesical position presence of a cystic mass with a 2.5 cm solid tumour inside	Not reported/*	Fibroepithelial tumour/NED (one year after surgery)
Son et al (2004)	39. Urinary retention and lower abdominal discomfort	CT: 14.5 × 12 cm heterogeneous soft tissue density mass located posterior to the UB and anterior to the rectum	16 × 13.5 × 8.5 cm tumour mass and a 5.1 × 3.3 × 1.5 cm tissue separated from the base of the mass/*Ω	Phyllodes tumour/NED 12 months after surgery and radiotherapy
Hoshi et al (2006)	70. General fatigue, lower abdominal pain	MRI: mass in the SV with a thin capsule of low-signal intensity; with compression of the prostate to the left anterior side but distinct from the prostate	4.5 cm in diameter, coarsely lobulated tumour with a smooth surface and surrounded by a thin fibrous capsule/°	Epithelial stromal tumour with phyllodes tumour-like features/NED 14 months after surgery
Lee et al (2006)	46. Asymptomatic	Sagittal T2-wighted MRI: multiseptated cystic lesion with heterogeneous signal intensity, originating from the posterior region of the prostate and extending superiorly over the UB	7.5 × 7 × 6 cm, well-circumscribed, oval, rubbery and lobulated contour mass/@	Cystadenoma/NED 6 months after surgery

^ In the cystomyoma of Plaut and Standard it is unclear whether the epithelium is a component of the neoplasm or an entrapped native structure. For these reasons the neoplasm should not be considered as a true MEST.

Treatment: * tumorectomy Ω left vesciculectomy @ Removal tumour mass, left SV, portion of both right and left vas deferens ° radical cysto-prostatectomy # radical cystoprostatectomy and low anterior resection of the rectum. = tumorectomy, removal of a portion of the bladder, of both right and left vas deferens and rectal muscularis propria.

**Legend: **UB = Urinary bladder; NED = No evidence of disease; IVP = Excretory urography; US = Ultrasonography; SV = Seminal vesicle.

## Conclusion

SVC and MEST are two distinct pathological entities with different histological features and clinical outcome. Predicting the potential biological behaviour of MEST remains difficult because accurate prognostic parameters are not available. In our case MRI was able to avoid an exploratory laparotomy and to establish an accurate conservative surgical treatment of the tumour.

## List of abbreviations

MEST: mixed epithelial and stromal tumour; US: ultrasound; WHO: World Health Organization; CT: Computed tomography; MRI: magnetic resonance imaging; CEA: carcinoembryonic antigen; PAP: prostate acid phosphatase; PSA: prostate specific antigen.

## Consent

Written informed consent was obtained from the patient for publication of this case report.

## Competing interests

The authors declare that they have no competing interests.

## Authors' contributions

IF collected the tissue specimen, made the histological diagnosis and images, revised the manuscript. IP made the radiological diagnosis, prepared radiological images. BM conceived the study and revised the manuscript.  ML, FF, GP: collected clinical records and wrote the manuscript. All authors read and approved the final manuscript.  
